# Accumulation of LOX-1^+^ PMN-MDSCs in nasopharyngeal carcinoma survivors with chronic hepatitis B might permit immune tolerance to epstein–barr virus and relate to tumor recurrence

**DOI:** 10.18632/aging.202149

**Published:** 2020-12-03

**Authors:** Xing Li, Jin-Long Li, Nan Jiang, Jie Chen, Zi-Ming Liang, Zhen-Lin Zhao, Yan-Fang Xing

**Affiliations:** 1 Shenzhen Ruipuxun Academy for Stem Cell and Regenerative Medicine, Shenzhen 518118, People’s Republic of China; 2 Department of Medical Oncology and Guangdong Key Laboratory of Liver Disease Research, The Third Affiliated Hospital of Sun Yat-sen University, Guangzhou 510630, People’s Republic of China; 3 Institute of Biotherapy, School of Laboratory Medicine and Biotechnology, Southern Medical University, Guangzhou 510515, People’s Republic of China; 4 Department of Transplantation, The Second Affiliated Hospital of Southern University of Science and Technology and the Third People’s Hospital of Shenzhen, Shenzhen 510623, People's Republic of China; 5 Department of Nephrology, The Third Affiliated Hospital of Guangzhou Medical University, Guangzhou 510150, People’s Republic of China

**Keywords:** Epstein-Barr virus, lectin-type oxidized LDL receptor-1, polymorphonuclear myeloid-derived suppressor cell, nasopharyngeal carcinoma, chronic hepatitis B

## Abstract

Chronic hepatitis B (CHB) has been reported to be associated with impaired prognosis for patients with nasopharyngeal carcinoma (NPC). However, the latent mechanism is unclear. Polymorphonuclear myeloid-derived suppressor cells (PMN-MDSCs) induce immune suppression in CHB and promote the development of hepatocellular carcinoma. Lectin-type oxidized LDL receptor-1 (LOX-1) was recently identified as a specific marker for PMN-MSDC. We found NPC survivors with CHB had high levels of LOX-1^+^ PMN-MDSCs. LOX-1^+^ PMN-MDSCs significantly reduced T cell proliferation and activation. Endoplasmic reticulum stress was induced in LOX-1^+^ PMN-MDSCs. In addition, LOX-1^+^ PMN-MDSCs increased their expression of NOX2, a key reactive oxygen species (ROS)-related genes, and levels of ROS illustrated by the DCFDA test. The ROS inhibitor N-acetylcysteine abrogated the suppression of LOX-1^+^ PMN-MDSCs on T cell activation. The EBV DNA-positivity rate was higher in NPC survivors with CHB than in NPC patients without CHB. Those presenting with positive EBV DNA displayed higher LOX-1^+^ PMN-MDSC levels. LOX-1^+^ PMN-MDSCs suppressed the CD8^+^ T cell response against EBV. This study revealed LOX-1^+^ PMN-MDSC accumulation and activation in NPC survivors with CHB. LOX-1^+^ PMN-MDSCs might suppress the host immune response to EBV through ER stress/ROS pathway. These results explained the association of CHB with unfavorable NPC prognosis.

## INTRODUCTION

Nasopharyngeal carcinoma (NPC) has an unbalanced geographical global distribution and is one of the major malignancies in East and Southeast Asia [1]. NPC is highly sensitive to ionizing radiation [1]; however, it has the highest risk of distant metastasis among all head and neck cancers and a high local recurrence of 10%–19% [2]. Thus, the mechanism of NPC tumor recurrence is receiving close attention in this field [3]. Non-keratinizing cancer is the major pathological subtype of NPC in epidemic areas (95%) and is predominantly associated with Epstein–Barr virus (EBV) infection [1], which is perhaps the most common causal agent of NPC [4]. EBV DNA is considered a biomarker for NPC development [5] and recurrence after radical treatments [1, 4, 6]. Notably, hepatitis B virus (HBV) is another virus related to NPC [7–9], and in the last decade has also reached epidemic levels in East and Southeast Asia [10]. Our previous study found that HBV infection was associated with impaired prognosis of NPC patients [11]. However, the latent mechanism remains unclear.

Chronic hepatitis B (CHB) is a chronic inflammatory disease with immune system dysfunction [12]. Recent studies have found that polymorphonuclear myeloid-derived suppressor cells (PMN-MDSCs) play a critical role in CHB [13–15]. PMN-MDSCs are critical immunosuppressive cells in inflammation and malignancies, which suppress immune cell activation and induce immune tolerance [16, 17]. PMN-MDSCs accumulate in CHB patients and suppress virus-specific cytotoxic T cells [13]. However, PMN-MDSCs are reported to promote the development of hepatocellular carcinoma (HCC) [18, 19], which is a consequence of CHB. These results indicated that PMN-MDSCs might promote NPC recurrence among NPC survivors with CHB.

Recent studies, including ours, found that lectin-type oxidized LDL receptor-1 (LOX-1) is a specific marker for human PMN-MDSCs, which leads to standardization and simplification of PMN-MDSC testing [17, 18, 20]. LOX-1^+^CD15^+^ cells in whole blood of humans were defined as LOX-1^+^ PMN-MDSCs, with LOX-1^-^CD15^+^ considered as normal neutrophils (PMNs) [17, 18, 20]. We speculated that LOX-1^+^ PMN-MDSCs may accumulate in NPC survivors with CHB, suppress EBV-specific T cell responses, and facilitate NPC recurrence. In the present study, we investigated the existence and suppressive function of LOX-1^+^ PMN-MDSCs in NPC survivors with CHB as well as their clinical significance.

## RESULTS

### LOX-1^+^ PMN-MDSCs were elevated in NPC survivors with CHB

**Figure 1:**
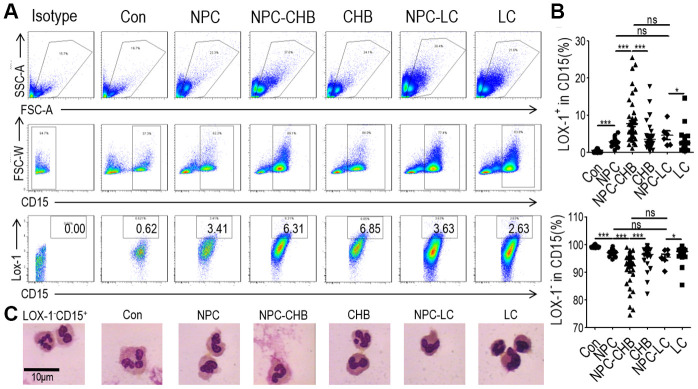
**Accumulation of LOX-1^+^ PMN-MDSCs in NPC survivors.** (**A**) Gating strategy and statistical analysis of LOX-1^+^ PMN-MDSCs by flow cytometry analysis in NPC survivors with CHB (NPC-CHB), NPC survivors with LC (NPC-LC), and their control, including NPC survivors without CHB, patients with CHB and patients with LC, and healthy donors (Con). (**B**) Typical morphology of sorted LOX-1^+^ PMN-MDSCs from each group and LOX-1^−^PMN. Abbreviations: PMN-MDSC, polymorphonuclear myeloid-derived suppressor cell; NPC, nasopharyngeal carcinoma; CHB, chronic hepatitis B; LC, liver cirrhosis.

During the period between June 2017 and May 2020, we investigated 56 NPC survivors with CHB. Among them, 45 survivors initiated entecavir/tenofovir before or during radical treatment. Six patients presented with compensatory cirrhosis diagnosed by ultrasound with Child–Pugh Score A liver function. Among the 50 survivors without cirrhosis, five patients had a slightly elevated alanine aminotransferase (ALT) level (> 40 U/L), which were all below 2 ULN, while the others presented with normal ALT. All patients received regular liver function surveillance, which displayed stable ALT levels for at least 6 months after enrollment. To identify the LOX-1^+^ PMN-MDSCs among NPC survivors with CHB, NPC survivors without CHB, healthy controls, patients with CHB, and liver cirrhosis patients were used as controls. LOX-1^+^ PMN-MDSCs were less than 1% of alive cells in whole blood in healthy controls. NPC survivors without CHB and patients with CHB had a significantly higher LOX-1^+^ PMN-MDSC frequency than healthy controls. NPC survivors with CHB had the highest level of LOX-1^+^ PMN-MDSCs among all the groups. However, NPC survivors with liver cirrhosis due to CHB did not have higher LOX-1^+^ PMN-MDSC levels than NPC survivors without CHB, though their LOX-1^+^ PMN-MDSC frequency was higher than that of liver cirrhosis patients. LOX-1^-^CD15^+^ cells (PMNs) frequency decreased accordingly. (Figure 1A, 1B). Morphologically, LOX-1^+^ PMN-MDSCs presented largely band-shaped nuclei while LOX-1^-^CD15^+^ PMNs presented a polymorphonuclear type. The morphology of LOX-1^+^ PMN-MDSCs between groups was similar (Figure 1C). Overall, the counts of LOX-1^+^ PMN-MDSCs were elevated among NPC survivors with CHB. CHB played a critical role in the accumulation of LOX-1^+^ PMN-MDSCs among NPC survivors with CHB.

### LOX-1^+^ PMN-MDSCs of NPC survivors with CHB suppressed T cell proliferation and activation

**Figure 2:**
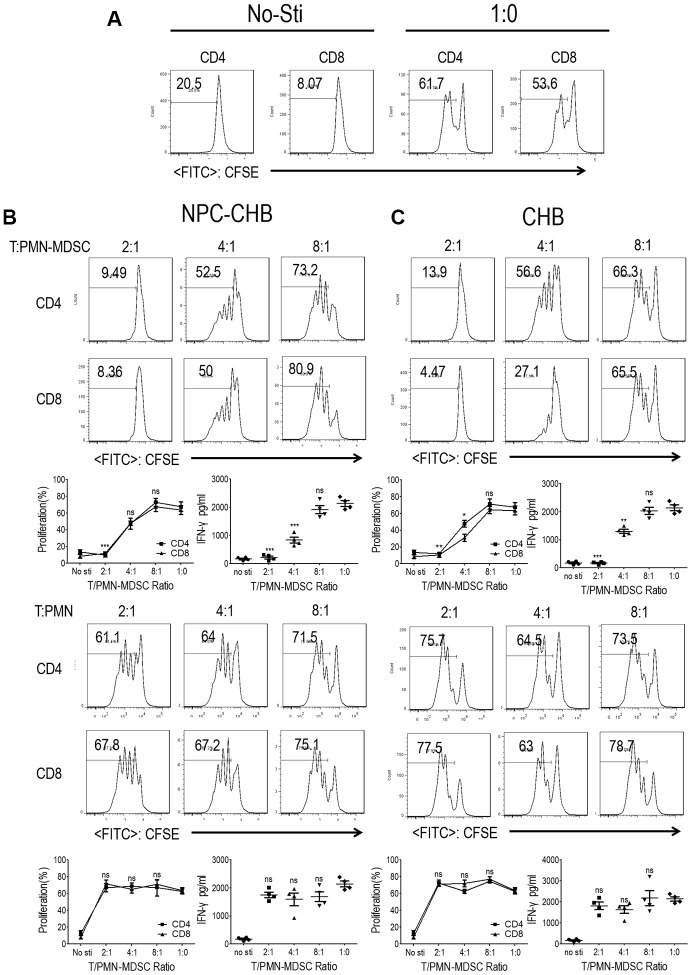
**LOX-1^+^ PMN-MDSCs from NPC survivors with CHB and from patients with CHB suppressed T cell proliferation and activation.** CD3^+^ T cells from PBMCs were labeled with CFSE, stimulated with anti-CD3 and anti-CD28, and then cocultured with LOX-1^+^ PMN-MDSCs or LOX-1^-^ PMN from the same donors at different ratios for 3 days. CD4^+^ and CD8^+^ T cell proliferation was evaluated by CFSE labeling and IFN-γ production in supernatants by ELISA. (**A**) T cells receiving no anti-CD3/anti-CD28 stimulation were used as negative controls with stimulated T cells without the addition of LOX-1^+^ PMN-MDSC/PMN as a positive control. (**B**) The influence of LOX-1^+^ PMN-MDSCs or LOX-1^-^ PMN from NPC survivors with CHB on T cell proliferation was displayed by representative of one independent experiment, cumulative data, and concentration of IFN-γ in the media. (**C**) The influence of LOX-1^+^ PMN-MDSCs or LOX-1^-^ PMN from CHB patients on T cell proliferation was shown by representative of one independent experiment, cumulative data, and concentration of IFN-γ in the media. (Comparison was made between the 1:0 group and experimental groups), (n = 4 in each group) *, *P* < 0.05; **, *P* < 0.01; ***, *P* < 0.001. Abbreviations: PMN-MDSC, polymorphonuclear myeloid-derived suppressor cell; NPC, nasopharyngeal carcinoma; CHB, chronic hepatitis B; PBMC, peripheral blood mononuclear cells; PMN, polymorphonuclear cell; CFSE, 5,6-carboxyfluoresceindiacetate, succinimidylester; IFN, interferon.

The suppressive ability of T cell proliferation and activation is a key characteristic of PMN-MDSCs [16]. To confirm the immunosuppressive capacity of LOX-1^+^ PMN-MDSCs in NPC survivors with CHB, T cells and LOX-1^+^ PMN-MDSCs from NPC survivors with CHB were purified from whole blood using flow sorting, with cells from patients with CHB as controls. LOX-1^-^CD15^+^ PMNs were used as negative controls. CFSE-labeled peripheral blood mononuclear cells (PBMC)-derived CD3^+^ homologous T cells were stimulated with anti-CD3/anti-CD28 with the indicated ratio of LOX-1^+^ PMN-MDSCs or LOX-1^-^CD15^+^ PMN. T cells receiving no anti-CD3/anti-CD28 were used as negative controls with stimulated T cells without the addition of LOX-1^+^ PMN-MDSC/PMN as a positive control. (Figure 2A) Addition of LOX-1^+^ PMN-MDSCs from NPC survivor with CHB, resulted in significantly reduced proliferation of both CD4^+^ and CD8^+^ T cells in a dose-dependent manner. IFN-γ levels in the media were tested using ELISA, which showed that IFN-γ secretion decreased after the administration of LOX-1^+^ PMN-MDSCs. LOX-1^-^CD15^+^ PMNs showed no suppressive function (Figure 2B). Similarly, LOX-1^+^ PMN-MDSCs from CHB patients also presented immunosuppressive functions (Figure 2C), indicating that CHB played a critical role in the immune-suppressive capability of LOX-1^+^ PMN-MDSCs among NPC survivors with CHB.

### Endoplasmic reticulum (ER) stress mediates LOX-1^+^ PMN-MDSCs from NPC survivors with CHB

**Figure 3:**
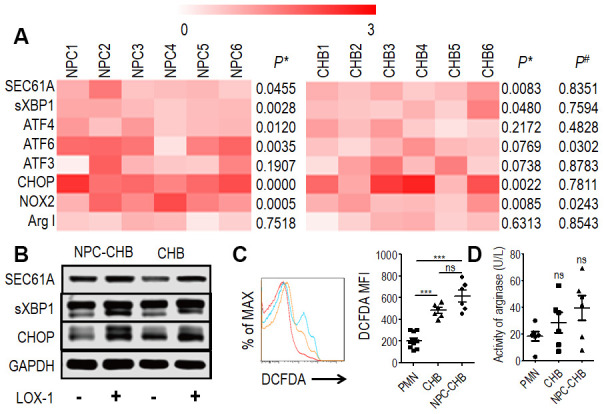
**Mechanism of LOX-1^+^ PMN-MDSCs from NPC survivors with CHB and from patients with CHB.** (**A**) Expression of *SEC61A*, s*XBP1*, *ATF4*, *ATF6*, *ATF3*, and *CHOP* in LOX-1^+^ PMN-MDSCs compared with LOX-1^-^PMN from the same donor were tested by RT-qPCR as well as *NOX2* and *ArgI*, (n = 6). P*, Expression of genes in LOX-1^+^ PMN-MDSCs compared with LOX-1^-^ PMN from the same donor. *P*^#^, Relative expression of genes in LOX-1^+^ PMN-MDSCs from NPC survivors with CHB compared with those from CHB patients. (**B**) Western blot analysis of expression of SEC61A, sXBP1, and CHOP proteins in LOX-1^+^ PMN-MDSCs compared with LOX-1^-^ PMN among NPC survivors with CHB and patients with CHB. (**C**) ROS level illustrated by DCFDA in LOX-1^+^ PMN-MDSCs from NPC survivors with CHB and patients with CHB. LOX-1^-^PMN from these patients was used as a control. Left: Representative flow cytometry data (red, LOX-1^-^PMN; yellow, LOX-1^+^ PMN-MDSCs from patients with CHB; blue, LOX-1^+^ PMN-MDSCs from NPC survivors with CHB). Right: Cumulative data (n = 6 in each group). (**D**) Activity of arginase was tested in LOX-1^+^ PMN-MDSCs from NPC survivors with CHB and patients with CHB. LOX-1^-^PMN from these patients was used as a control (n = 6 in each group). Abbreviations: PMN-MDSC, polymorphonuclear myeloid-derived suppressor cell; NPC, nasopharyngeal carcinoma; CHB, chronic hepatitis B; CHOP, CCAAT/enhancer binding protein; PMN, polymorphonuclear cell; ROS, reactive oxygen species; DCFDA, 2′,7′-dichlorofluorescein diacetate.

Previous reports indicated that ER stress is the key mechanism in regulating PMN-MDSCs [18, 20] thus, RT-qPCR was conducted to analyze LOX-1^+^ PMN-MDSCs from NPC survivors with CHB and patients with CHB. LOX-1^-^CD15^+^ PMNs from the same donor were used as controls. Expression of ER stress-related genes, including *SEC61A*, *sXBP1*, *ATF4*, *ATF6*, *ATF3*, and CCAAT/enhancer binding protein (*CHOP*), was increased in LOX-1^+^ PMN-MDSCs compared with LOX-1^-^CD15^+^ PMNs from the same donor among NPC survivors with CHB. The mRNA expression levels of *SEC61A,* s*XBP1*, and *CHOP* were elevated in LOX-1^+^ PMN-MDSCs from CHB patients. In addition, the expression of *ATF6* was higher in LOX-1^+^ PMN-MDSCs from NPC survivors with CHB compared with those from patients with CHB (Figure 3A). Western blot analysis confirmed that the expression of SEC61A, sXBP1, and CHOP proteins in LOX-1^+^ PMN-MDSCs was higher than that in LOX-1^-^CD15^+^ PMNs from both NPC survivors with CHB and patients with CHB (Figure 3B). Thus, ER stress was upregulated in LOX-1^+^ PMN-MDSCs from NPC survivors with CHB. CHB played a partial role in this mechanism.

### ROS production mediated the immune-suppression function of LOX-1^+^ PMN-MDSCs

**Figure 4:**
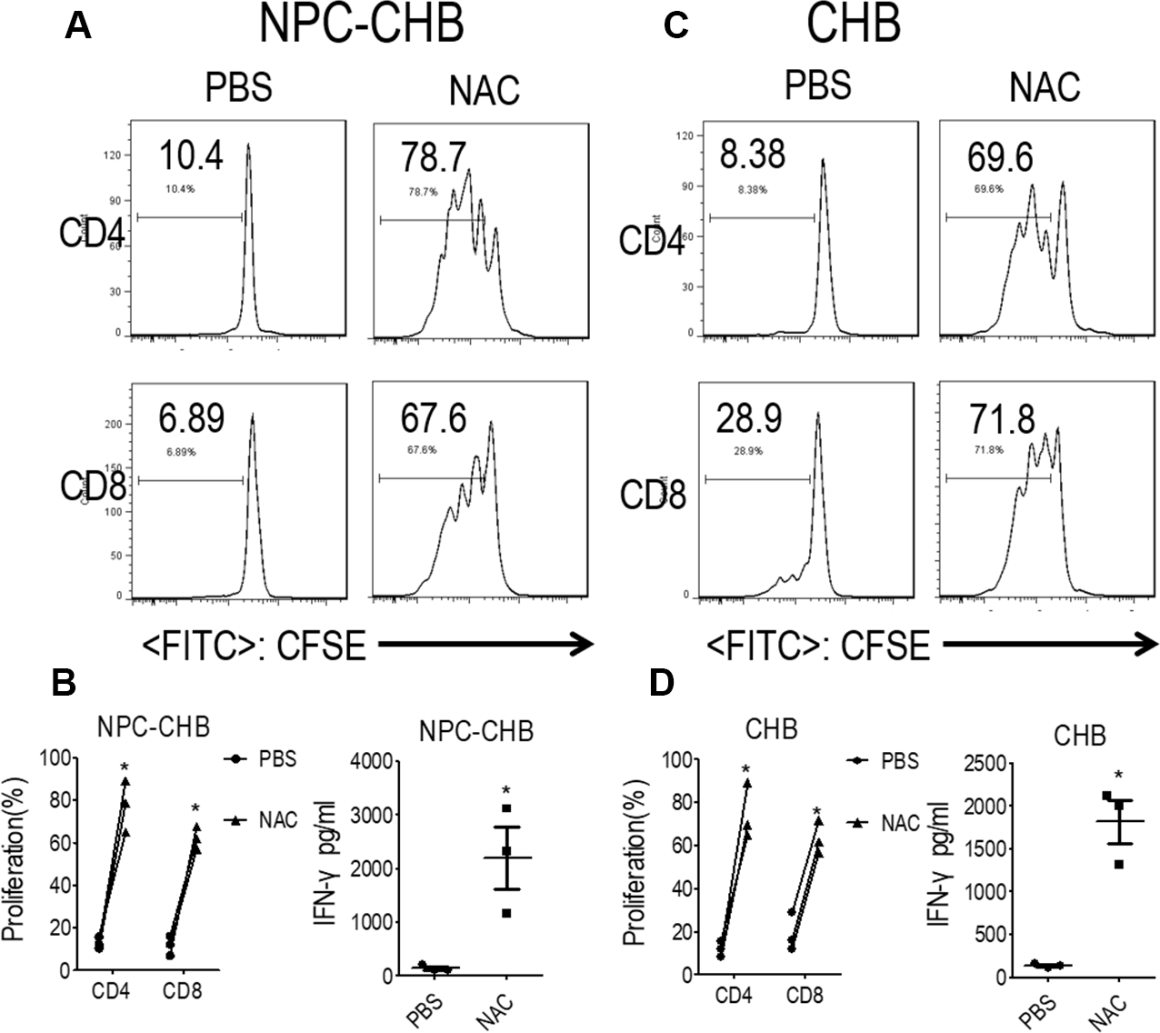
**LOX-1^+^ PMN-MDSCs suppressed functional T cells in a ROS-dependent manner.** (**A**, **B**) Effect of ROS inhibitor NAC on LOX-1^+^ PMN-MDSC function in NPC survivors with CHB. Autologous T cells were stimulated with anti-CD3 and anti-CD28, cocultured with LOX-1^+^ PMN-MDSCs from NPC survivors with CHB at a 2:1 ratio with NAC or PBS treatment. Evaluation of T cell proliferation by CFSE labeling and IFN-γ production in supernatants by ELISA. Representative flow cytometry data (A), cumulative data, and concentration of IFN-γ in the media (**B**) are shown (n = 3). (**C**, **D**) Effect of ROS inhibitor NAC on LOX-1^+^ PMN-MDSC function in CHB patients. Representative flow cytometry data (**C**), cumulative data, and concentration of IFN-γ in the media (**D**) are shown (n = 3)*, *P* < 0.05; ***, *P* < 0.001. Abbreviations: PMN-MDSC, polymorphonuclear myeloid-derived suppressor cell; ROS, reactive oxygen species; PMN, polymorphonuclear cell; NPC, nasopharyngeal carcinoma; CHB, chronic hepatitis B; CFSE, 5,6-carboxyfluoresceindiacetate diacetate; IFN, interferon.

PMN-MDSCs are reported to produce arginase I or ROS to suppress T cells [18, 20, 21]. The expression of *NOX2*, a key ROS-related gene in PMN-MDSCs, was investigated by RT-qPCR in LOX-1^+^ PMN-MDSCs from NPC survivors with CHB. LOX-1^+^ PMN-MDSCs from NPC survivors with CHB and those from CHB patients had higher *NOX2* mRNA levels compared with LOX-1^-^CD15^+^ PMNs (Figure 3A); To confirm these results, the DCFDA method was used to determine the ROS level of LOX-1^+^ PMN-MDSCs and demonstrated that LOX-1^+^ PMN-MDSCs from NPC survivors with CHB and patients with CHB had elevated levels of ROS (Figure 3C). In addition, RT-qPCR and arginase activity tests revealed that arginase I expression was not increased in LOX-1^+^ PMN-MDSCs compared with that in LOX-1^-^CD15^+^ PMNs (Figure 3A, 3D). To investigate this further, we added the ROS inhibitor NAC to the co-culture system. This abrogated the suppression of LOX-1^+^ PMN-MDSCs on T cell activation among NPC survivors with CHB and those from patients with CHB (Figure 4). Thus, ROS production mediated the immune-suppression function of LOX-1^+^ PMN-MDSCs from NPC survivors with CHB. CHB also contributed to a certain inductive role in the activation of LOX-1^+^ PMN-MDSCs in NPC survivors with CHB.

### LOX-1^+^ PMN-MDSCs may permit persistent EBV replication

**Figure 5:**
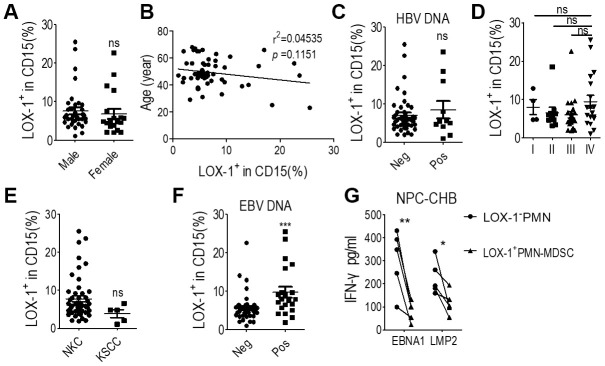
**LOX-1^+^ PMN-MDSCs may permit EBV replication in NPC survivors with CHB.** (**A**) LOX-1^+^ PMN-MDSC levels of NPC survivors of different genders; (**B**) Linear regression of LOX-1^+^ PMN-MDSC level with age; (**C**) LOX-1^+^ PMN-MDSC level of survivors with positive or negative serum HBV DNA results; (**D**) LOX-1^+^ PMN-MDSC level of survivors with different stages of NPC; (**E**) LOX-1^+^ PMN-MDSC level of survivors with different NPC pathological subtypes; (**F**) LOX-1^+^ PMN-MDSC level of survivors with positive or negative serum EBV DNA; (**G**) CD8^+^ T cell responses against the major antigenic EBV proteins, LMP2 and EBNA1, was demonstrated by changes in IFN-γ concentration. Abbreviations: PMN-MDSC, polymorphonuclear myeloid-derived suppressor cell; EBV, Epstein-Barr virus; NPC, nasopharyngeal carcinoma; CHB, chronic hepatitis B; ALT, alanine aminotransferase; KSCC, keratinizing squamous cell carcinoma; NKC, non-keratinizing carcinoma; IFN, interferon.

Clinicopathological analysis was conducted to evaluate the relationship between LOX-1^+^ PMN-MDSC levels and NPC prognosis. The level of LOX-1^+^ PMN-MDSC was not related to gender, age, HBV DNA status, NPC stages at diagnosis, or pathological subtypes among NPC survivors with CHB (Figure 5A–5E). Notably, the EBV DNA-positivity rate was higher in NPC survivors with CHB than in NPC patients without CHB (37.5% vs. 10%, *P* = 0.024). Those presenting positive EBV DNA displayed higher LOX-1^+^ PMN-MDSC levels (Figure 5F). Since EBV DNA is a strong prognostic factor for NPC recurrence [1, 6, 9], LOX-1^+^ PMN- MDSC may be involved in host immune tolerance to EBV.

To illustrate that LOX-1^+^ PMN-MDSCs permit EBV replication, T cells, LOX-1^+^ PMN-MDSCs, and LOX-1^-^CD15^+^ PMNs from NPC survivors with CHB were purified from whole blood using flow sorting. T cells were simulated with 2 μg/mL PepMix EBV peptide and anti-CD3, and co-cultured with LOX-1^+^ PMN-MDSCs or LOX-1^-^CD15^+^ PMN. After 72 h, ELISA testing of serum IFN-γ indicated that secretion of IFN-γ decreased after administration of LOX-1^+^ PMN-MDSCs compared with LOX-1^-^CD15^+^ PMNs (Figure 5G). Thus, LOX-1^+^ PMN-MDSCs suppress the immune elimination of EBV in NPC survivors.

## DISCUSSION

The mechanism of NPC recurrence is currently unclear. A series of risk factors are known to be predictive of tumor recurrence among NPC survivors, including tumor volume^22^, local invasion, histological subtype [22], pretreatment blood test [23], EBV DNA [24], etc. Among these, EBV DNA is considered one of the most significant prognostic factors, due to the etiological role of EBV in the development of NPC [4]. Recently, CHB was found to participate in the development of NPC and was identified as a prognostic factor [7–9, 11]. However, the specific role of CHB in the development of NPC is unclear. In the present study, we found that CHB promoted LOX-1^+^ PMN-MDSC expansion and activation in NPC survivors, and that LOX-1^+^ PMN-MDSCs may permit EBV immune tolerance. These results indicated that LOX-1^+^ PMN-MDSC mediated CHB induced NPC recurrence.

HBV may indirectly promote NPC recurrence. CHB is known to have a clear etiological role in the development of HCC [25]. In recent decades, CHB has been related to multiple malignancies, including lymphoma [26] and NPC [7–9, 11]. However, the role of CHB in the development of other malignancies remains controversial, indicating a possible indirect role in malignancies other than HCC. EBV has been confirmed to be a predictor for NPC diagnosis and recurrence in many studies, with its etiological role in NPC confirmed [1, 4, 6]. We found that LOX-1^+^ PMN-MDSCs in NPC survivors with CHB suppressed the CD8^+^ T cell response to EBV. PMN-MDSCs have been reported to promote immune tolerance of HBV [27, 28] and other infectious diseases [29]. Thus, LOX-1^+^ PMN-MDSCs in NPC survivors with CHB may permit immune tolerance to EBV and lead to tumor recurrence.

The induction mechanism of LOX-1^+^ PMN-MDSCs in NPC survivors shall be different from NPC patients. PMN-MDSC levels were reported to be elevated in NPC patients by several studies [30, 31]. EBV latent membrane protein 1 (LMP1) was reported to increase the production of IL-1β, IL-6, and GM-CSF in NPC tumor cells [30], which finally promoted MDSC in the tumor microenvironment. COX2 expression in tumor cells has been reported to promote MDSC expansion [31]. Notably, PMN-MDSC was minimal in malignances with lower tumor burden [18, 20]. And, NPC cells were not supposed to be abundant and active in NPC survivors who achieved complete tumor remission. Thus, the accumulation of LOX-1^+^PMN-MDSC in NPC survivors could not be assumed to be EBV activated NPC tumor cells. We found that NPC survivors presented higher LOX-1^+^ PMN-MDSC than healthy control, which might be induced by chronic inflammation due to radiation damage since PMN-MDSC could accumulated in patients with chronic inflammation [17, 27]. Chronic hepatitis B (CHB) patients presented higher PMN-MDSC which was consistent with previous study. Notably, NPC survivors with CHB presented significantly higher LOX-1^+^ PMN-MDSC than NPC survivors, which strongly indicated that CHB played a critical role in the accumulation of LOX-1^+^ PMN-MDSC in NPC survivors with CHB. In order to further support this speculation, we investigated the mechanism, LOX-1^+^PMN-MDSC in NPC survivors with CHB presented similar features with those from CHB patients, which indicated their similar origin. To the best of our knowledge, MDSC accumulation and activation among NPC survivors has not been reported before. Thus, EBV infection might not be the major cause of accumulation and activation of LOX-1^+^ PMN-MDSCs among NPC survivors.

Accumulation and activation of LOX-1^+^ PMN-MDSCs among NPC survivors with CHB might be due to multiple reasons, among which CHB played a critical role [13]. PMN-MDSC accumulation and activation has been reported in patients with inactive CHB [13], and CHB among NPC survivors was also inactive. Additionally, ER stress, as a key regulator of LOX-1^+^ PMN-MDSC [18, 20], exists in LOX-1^+^ PMN-MDSCs from NPC survivors with CHB as well as in patients with CHB. LOX-1^+^ PMN-MDSCs from both groups secreted ROS, which also supported their similar origin. Additionally, chronic inflammation due to radiation damage might also lead to LOX-1^+^ PMN-MDSC accumulation and activation among NPC survivors. NPC survivors without CHB also had slightly higher LOX-1^+^ PMN-MDSC levels than healthy controls, and chronic inflammation was reported to induce MDSC as well [27, 29]. Thus, chronic inflammation due to CHB, and also damage due to radiology treatment, can promote the increase in LOX-1^+^ PMN-MDSC abundance in NPC survivors with CHB.

A limitation of this study is that the LOX-1^+^ PMN-MDSC testing time after completion of anti-cancer therapy was not parallel among the NPC survivors. Additionally, the patient size was not large enough to account for the difference in tumor recurrence with different levels of LOX-1^+^ PMN-MDSC. The role of LOX-1^+^ PMN-MDSCs on EBV immune tolerance requires further evidence for a solid conclusion. The role of liver cirrhosis in the induction of LOX-1^+^ PMN-MDSCs among NPC survivors is still unclear due to the limited sample size. Monocytic MDSC was another important subtype of MDSC. This study did not investigate M-MDSC. However, its specific marker has not been identified yet. Its role in the NPC survivors needs further investigation.

This study revealed LOX-1^+^ PMN-MDSC accumulation and activation in NPC survivors, especially those with CHB. LOX-1^+^ PMN-MDSCs might suppress the host immune response to EBV through ER stress/ROS pathway. CHB played a critical role in the induction of LOX-1^+^ PMN-MDSC in NPC survivors with CHB. These results explained the association of CHB with unfavorable NPC prognosis.

## MATERIALS AND METHODS

### Patients and healthy donors

NPC survivors were defined as NPC patients who achieved complete regression (CR) after completion of radical treatment. NPC survivors who had completed anti-cancer treatment for more than 3 months were enrolled in this study. During the period between June 2017 and May 2020, we investigated 56 NPC survivors with CHB, 20 NPC survivors without CHB, 50 patients with inactive chronic hepatitis B (alanine aminotransferase level below two times the upper limit of normal, ULN) under nucleoside analog treatment, 50 patients with liver cirrhosis due to hepatitis B, and 50 healthy controls in the Third Affiliated Hospital of Guangzhou Medical University, the Third Affiliated Hospital of Sun Yat-sen University, and the Third People’s Hospital of Shenzhen. The diagnosis of NPC was confirmed by pathology. All patients and healthy controls were also screened for serum human immunodeficiency virus antibody, hepatitis B surface antigen, hepatitis C virus antibody, hepatitis D virus (HDV) antigen, and HDV antibody. Patients and healthy controls who were positive for HIV, chronic hepatitis virus infection other than HBV and other acute infections (including pneumonia, urinary tract infection, etc.), who were pregnant, who had received systematic corticosteroids or immunosuppressive agents, or had fever or alternate malignancies were excluded from this study. This study was approved by the Clinical Ethics Review Board of the Third Affiliated Hospital of Guangzhou Medical University, the Third Affiliated Hospital of Sun Yat-sen University, and the Third People’s Hospital of Shenzhen. Written informed consent was obtained from all patients at the time of admission.

### Flow cytometric analysis

Blood samples were analyzed within 6 h of sampling. Whole blood was treated with erythrocyte lysate (Tonbo Biosciences, USA). Anti-human antibodies CD15-eFluor450, CD15-fluorescein isothiocyanate (FITC), and LOX-1-APC and their corresponding isotype controls were purchased from Biolegend. The cell phenotypes were analyzed by flow cytometry on a FACSAria II flow cytometer (BD Bioscience), and data was analyzed using FlowJo V10.0.7 (FlowJo, OR, USA). For flow cytometric sorting, a BD FACSAria cell sorter (BD Bioscience) was used. The strategy for MDSC sorting was LOX-1^+^CD15^+^ for PMN-MDSCs and LOX-1^-^CD15^+^ for PMNs from live whole blood cells.

### T cell proliferation and activation assays

T cell proliferation was determined by a CFSE (5,6-carboxyfluoresceindiacetate, succinimidylester) dilution assay. Purified T cells were stained with CFSE (3 μM; Invitrogen), stimulated by 3-h pre-coating with 0.5 μg/ml anti-CD3 and 0.5 μg/ml anti-CD28 (eBioscience), and cultured alone or co-cultured with autologous PMN-MDSCs/PMNs at the indicated ratios for 72 hours. The cells were then labeled for surface marker expression with CD4-PE or CD8-PE-Cy5 antibodies, and T cell proliferation was analyzed using a flow cytometer. All cultures were carried out in the presence of 20 IU/ml recombinant human IL-2 (PeproTech, Rocky Hill, USA) in RPMI 1640 (Life Technologies) for 72 h at 37° C. Where indicated 1 mM N-acetylcysteine (NAC, Sigma), a reactive oxygen species (ROS) inhibitor, was added to the culture system on day 0.

### CD8^+^ T cell activation by antigenic EBV proteins: LMP2 and EBNA1

CD8^+^ T cells, LOX-1^+^ PMN-MDSCs, and LOX-1^-^CD15^+^ PMNs from NPC survivors with CHB were purified using flow sorting. T cells were simulated with 0.5 μg/ml 3-h pre-coated anti-CD3 and 2 μg/mL of PepMix EBV peptide (EBNA1 or LMP2; JPT, USA) in the presence of 20 IU/ml recombinant human IL-2 in RPMI 1640 for 72 hours at 37° C and cocultured with 2:1 LOX-1^+^ PMN-MDSCs or LOX-1^-^CD15^+^ PMN. Conditioned medium was collected for ELISA.

### Measurement of intracellular ROS levels

ROS measurements were determined using 2′,7′-dichlorofluorescein diacetate (DCFDA) staining. Briefly, whole blood was incubated with 2.5 μM DCFDA at 37° C for 30 min after red blood cell lysis. Cells were then washed and resuspended in phosphate-buffered saline (PBS), stained with CD15-eFluor450 and LOX-1-APC at 4° C for 30 min. ROS was analyzed using flow cytometry with excitation and emission wavelengths of 490 and 520 nm, respectively, in LOX-1^+^CD15^+^ cells and LOX-1^-^CD15^+^ cells.

### ELISA

Culture supernatants of T cells and LOX-1^+^ PMN-MDSC or PMN co-culture systems were collected for ELISA testing. Interferon-γ (IFN-γ) quantification in culture supernatants was determined by an enzyme-linked immunosorbent assay (ELISA) following the manufacturer’s instructions (DKW12-1000-09, Dakewe Bioengineering Co., Shenzhen, Guangdong).

### Arginase activity assay

The activity of arginase was measured in cell lysates. Briefly, cells were lysed with 0.1% Triton X-100 for 30 min, then 25 mM Tris-HCl and 10 mM MnCl_2_ were added. The enzyme was activated by heating at 56° C for 10 min. Arginine hydrolysis was performed by incubating the lysate with 0.5 M L-arginine for 120 min at 37° C. After the addition of α-isonitrosopropiophenone (dissolved in 100% ethanol), the urea concentration was measured at 540 nm, followed by heating at 95° C for 30 min.

### Quantitative reverse transcription PCR (RT-qPCR)

RNA was extracted with a Multisource Total RNA Miniprep Kit (AXYGEN, CA, USA), RT-qPCR was performed using commercially available primers and SYBR Premix Ex Taq II (Code, DRR081; Takara Biotechnology (Dalian) Co., Ltd., Dalian, China). The fluorescence for each cycle was quantitatively analyzed using the ABI Prism 7000 sequence detection system (Life Technologies). The results are reported as relative expression, normalized to the β-actin housekeeping gene as an endogenous control and expressed in arbitrary units. Primers used are listed in Supplementary Table 1.

### Evaluation of EBV DNA levels

Peripheral venous blood (3 mL) was collected from NPC survivors into EDTA-containing tubes and centrifuged at 3,000 × g for 5 min. Total plasma DNA was extracted using a QIAamp DNA Blood Mini Kit (Qiagen, Hilden, Germany). Fluorescence polymerase chain reaction (PCR) was carried out using an EBV PCR quantitative diagnostic kit (Da-An Genetic Diagnostic Center, Guangzhou, China) targeting the BamHI-W region of the EBV genome. Data were analyzed using the Applied Biosystems 7300 SDS software (Beijing, China).

### Patient follow-up and statistical analyses

Patients returned for follow-up appointments at least every 3–6 months for a liver function test thereafter until death or last follow-up. Variables in different groups were compared using the χ² test (or Fisher's exact test, if indicated) and t-test or nonparametric Mann–Whitney U tests. The criterion for statistical significance was set at α = 0.05, and all *P*-values were based on two-sided tests. For *in vitro* experiments, statistical analyses were performed using paired t-tests. Comparison of parameters between groups of patients was conducted using nonparametric Mann–Whitney U tests. Correlations between different parameters were analyzed using the Spearman rank test. Statistical tests were performed using GraphPad Prism version 5.0a and SPSS Statistics 20.0. *P*-values of 0.05 were considered significant.

### Ethics statement

This study was approved by the Clinical Ethics Review Board of the Third Affiliated Hospital of Guangzhou Medical University, the Third Affiliated Hospital of Sun Yat-sen University and the Third People’s Hospital of Shenzhen. A written informed consent was obtained from all the patients at the time of admission.

## Supplementary Material

Supplementary Table 1
