# Supplementary material for: Accumulation of LOX-1+ PMN-MDSCs in nasopharyngeal carcinoma survivors with chronic hepatitis B might permit immune tolerance to epstein–barr virus and relate to tumor recurrence
Source: Aging (Albany NY). 2020 Dec 3;13(1):437–49. doi: 10.18632/aging.202149 (PMC7834990; doi:10.18632/aging.202149)
Supplement: Supplementary Table 1 [file aging-13-202149-s001.pdf]

Supplementary Table 1. Primers used.

NOX2 for	5’-TCGAAATCTGCTGTCCTTCC-3’
NOX2 rev	5’-TATTGACTCGGGCATTCACA-3’
NOX2 for	5’-TCGAAATCTGCTGTCCTTCC-3’
β-actin for	5’-CTCCATCCTGGCCTCGCTGT-3’
β-actin rev	5’-GCTGTCACCTTCACCGTTCC-3’
ARGI for	5’-CTTGTTTCGGACTTGCTCGG-3’
ARGI rev	5’-CACTCTATGTATGGGGGCTTA-3’
DDIT3 (CHOP) for	5’-GCACCTCCCAGAGCCCTCACTCTCC-3’
DDIT3 (CHOP) rev	5’- GTCTACTCCAAGCCTTCCCCCTGCG-3’
ATF3 for	5’-TGCCTCGGAAGTGAGTGCTT-3’
ATF3 rev	5’-GCAAAATCCTCAAACACCAGTG-3’
GADPH for	5’- GGAGTCAACGGATTTGGTCGTA-3’
GADPH rev	5’- GGCAACAATATCCACTTTACCAGAGT-3’
sXBP-1 for	5’-CTGAGTCCGCAGCAGGTG-3’
sXBP-1 rev	5’-AGTTGTCCAGAATGCCCAACA-3’
ATF4 for	5’ATGACCGAAATGAGCTTCCTG-3’
ATF4 rev	5’-GCTGGAGAACCCATGAGGT-3’
ATF6 for	5’-TCCTCGGTCAGTGGACTCTTA-3’
ATF6 rev	5’-CTTGGGCTGAATTGAAGGTTTTG-3’
SEC61A for	5’-TCATCCTGCCGGAAATTCAGA-3’
SEC61A rev	5’-AGGGTGATAGCGGTCCACA-3’
